# Evaluation of Microwave Applicator Design on Electromagnetic Field Distribution and Heating Pattern of Cooked Peeled Shrimp

**DOI:** 10.3390/foods10081903

**Published:** 2021-08-16

**Authors:** Érica S. Siguemoto, Jorge A. W. Gut, Georgios Dimitrakis, Sebastien Curet, Lionel Boillereaux

**Affiliations:** 1 Department of Chemical Engineering, Escola Politécnica, University of São Paulo, 05508-080 São Paulo, Brazil; erica.siguemoto@cirad.fr (É.S.S.); jorgewgut@usp.br (J.A.W.G.); 2 GEPEA, UMR CNRS 6144, Oniris, Université de Nantes, F-44000 Nantes, France; sebastien.curet@oniris-nantes.fr; 3 Food Research Center (FoRC), University of São Paulo, 055508-080 São Paulo, Brazil; 4 Department of Chemical and Environmental Engineering, University of Nottingham, Nottingham NG7 2RD, UK; Georgios.Dimitrakis@nottingham.ac.uk

**Keywords:** microwave, single-mode, modelling, heating uniformity, shrimp

## Abstract

Non-uniform temperature distribution within solid food is a major problem associated with microwave heating, which limits industrial applications. Therefore, an experimentally validated 3D model was proposed to study the effect of microwave applicator geometry on the electromagnetic field distribution and heating pattern of shrimp under different processing conditions. Simulation results were compared with physical experiments, in which a cooked peeled shrimp sample was heated using two different laboratory-scale microwave applicators (rectangular and cylindrical cavities). For the rectangular applicator, the temperature distribution within the shrimp, when examined in cross-section, was more homogeneous compared to that of the cylindrical applicator. The results showed the influence of the complex shape of the food on the temperature distribution during microwave heating, as well as of process parameters (input power and geometry cavity). Moreover, this modelling method could provide a better understanding of the microwave heating process and assist manufacturing companies to evaluate a suitable microwave applicator according to their specific purpose.

## 1. Introduction

The potential of novel food preservation technologies, such as electro-heating (ohmic, radiofrequency, and microwave), has been explored to provide a reliable and effective process for a sustainable global market [1]. Conceptually, thermal treatment of foods is designed to deliver fast and uniform heating, ensuring food safety and shelf-stability with high sensory and nutritional qualities [2]. When a solid food is heated by microwave energy, the prediction of the slowest or fastest heating zones is a challenging task from an engineering point of the view since the heating uniformity is influenced by intrinsic and extrinsic factors [3]. Temperature-dependent dielectric/thermal properties, heterogeneous electric field distribution, size, and geometry of the food are critical to the uniformity of the heating process [4,5].

Recent solutions have been proposed to reduce the non-uniformity of microwave heating in solid foods, such as movement along conveyor belts [6,7], use of turntables [8,9,10], use of water instead of air surrounding the food product [6,11], use of novel microwave generators [12,13,14,15], and use of alternative microwave frequencies [16,17]. However, the non-uniformity and complex heating patterns are still a challenge that requires detailed modelling of the field distribution in the cavity and food, as Knoerzer et al. [4] and Dinani et al. [15] stated. 

Due to the complexity of the equations coupling electromagnetic field propagation and heat transfer, the prediction of the dynamic temperature distribution becomes a labour-consuming task when the complex size and shape of the actual food under processing are taken into consideration [18,19]. Virtual visualisation of the microwave heating process by computational tools has been used to better understand electro-heating mechanisms and, consequently, to allow the design/improvement of microwave applicators [20,21]. Moreover, 3D computer simulation can explore how electromagnetic energy is dissipated within the food in order to maximise the microwave applicator efficiency and energy use [7,22]. 

In a processing system, the electromagnetic waves generated by a microwave power source are typically directed to a single-mode or a multi-mode cavity. For the design of a 2450 MHz single-mode cavity, its dimensions need to be proportional to the microwave half wavelength in one dimension, typically along the direction of propagation. In contrast, when the size of the cavity in at least two dimensions is a multiple of the half wavelength, superimposed standing wave patterns occur and the field distribution is complex, requiring methods of design optimisation and power coupling for proper prediction and application [6,23]. For a single-mode cavity operating conditions, the material load is placed at the location where maximum electromagnetic field intensity provides the best thermal processing performance, due to the focused microwave radiation and a more uniform and predictable field distribution [24,25]. 

**Figure 1:**
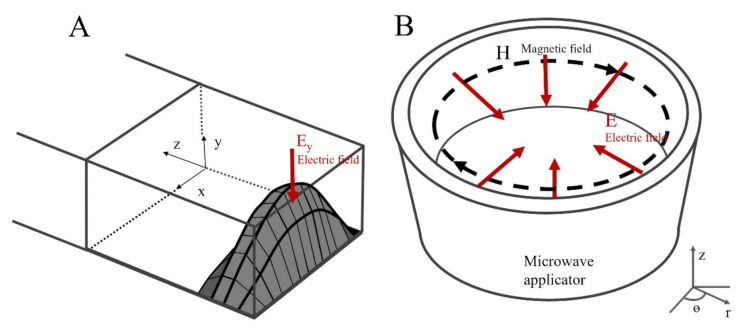
Representation of the electric field patterns for a rectangular TE_010_ applicator (**A**) and cylindrical TM_010_ applicator (**B**).

Rectangular and cylindrical single-mode cavities allow the resonance of a single transverse electric mode (TE) or transverse magnetic mode (TM) at a particular frequency [26]. Fundamental TE and TM modes of empty rectangular and cylindrical applicators are shown in Figure 1A,B, respectively. As in the example for the TE_010_ mode (Figure 1A), the electric field in the *x*-direction has one semi-sinusoidal variation while is constant in the *y*-direction, with no component in the *z*-direction [27].

Recently, cylindrical cross-section cavities have been applied for industrial pasteurisation and sterilisation of pumpable food fluids in continuous flow [28,29,30,31]. Otherwise, rectangular cavities have been explored for microwave-assisted tempering [32], freezing [33], and also pasteurisation of solid foods [3,19,34]. 

Besides cavity design, intrinsic factors such as size, shape, and composition of the food influence the heating uniformity during microwave treatment. Wäppling Raaholt and Isaksson [5] discussed the local overheating phenomenon according to the different shape of foods from the simulated results of temperature distribution during the thawing process. For a rectangular food block, the intensity of the microwave energy at the corners and edges was reported as the main heating uniformity problem [32,35,36]. For sphere- or cylinder-shaped foods (i.e., food with convex surfaces), refraction and reflection phenomena explain the centre overheating phenomenon, when the food is placed in a turntable microwave oven [37,38,39]. Though these previous studies are useful to comprehend the microwave power absorption and temperature distribution inside food models, the simulations are limited to rectangular, cubic, or cylindrical foods that are different from real-life conditions. 

As a case study, cooked peeled shrimp was chosen herein due to the irregular geometry and small size, providing a challenge for the implementation of numerical modelling and the strategy of modelling-based product development. Moreover, the rationale for the food chosen is that this study is part of a larger collaborative project on microwave post-processing of cooked shrimp to extend the shelf-life and to ensure the safety. For that, the first step is to understand the influence of different microwave cavities, namely, cylindrical and rectangular single-mode applicators, on temperature distribution of cooked peeled shrimp to improve microwave heating performance.

## 2. Materials and Methods

### 2.1. Microwave Applicators 

Experiments for the heating of cooked peeled shrimp were carried out with two different setups: (1) a commercial microwave reactor (2450 MHz) with a cylindrical cavity resonating in the TM_10_ mode (Discover, CEM Corporation, Matthews, NC, USA), and (2) a custom-made WR340 rectangular guide with a progressive wave in the TE_10n_ mode at 2450 MHz.

**Figure 2:**
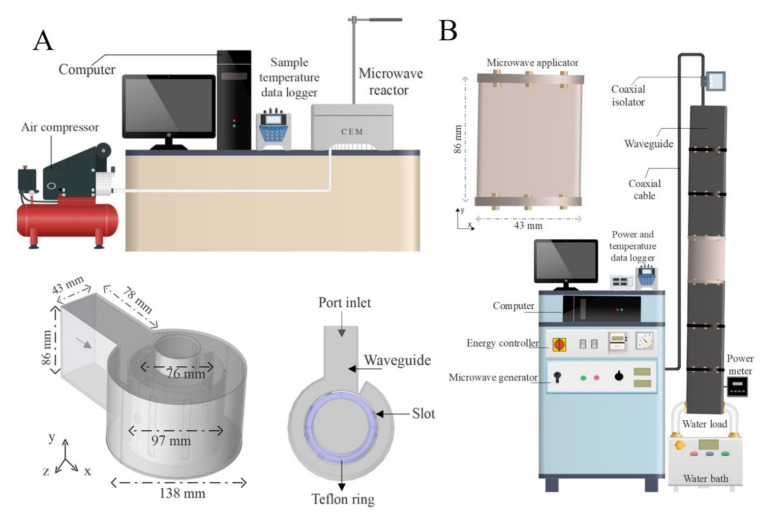
Schematic diagram of the experimental setup using the commercial CEM^®^ microwave system (**A**) and custom-made system (**B**).

In the microwave reactor from CEM Corporation (Matthews, NC, USA), the nominal microwave power generated by the magnetron (1 W ≤ P ≤ 300 W) propagates along a rectangular TE_10_ waveguide (86 × 43 × 78 mm), reaches a cylindrical cavity and goes through six inlet ports (73 mm of height and 7.4 mm of width) after a polytetrafluoroethylene (PTFE) ring and propagates to the cylindrical cavity (138 mm of outside diameter and 97 mm of inside diameter), where the sample was placed using a Pyrex glass support. There is a complex interaction between the cylindrical applicator and the magnetron, as described by Sturm et al. [40]. Microwave energy is partially absorbed by the load, and another part (i.e., reflected microwave energy) propagates forth and back within the applicator. By combining the measured dimensions of the microwave device and further descriptions of geometry from Damilos et al. [41], we modelled the microwave system in COMSOL Multiphysics (v. 5.5, COMSOL Inc., Stockholm, Sweden), as shown in Figure 2A.

The custom-made microwave apparatus consisted of a microwave generator (GMP03KSM, Sairem, Décines-Charpieu, France) with nominal microwave power P = 300 W that was connected to a brass WR340 rectangular waveguide (cross-section 86 × 43 mm) by a coaxial cable and waveguide transition. An element of this waveguide, where a shrimp was placed in the middle using a PTFE support, takes the place of a cavity where microwave energy was transmitted (z-direction) in TE_10n_ mode (86 × 43 × 200 mm). After passing through the applicator, the remaining forward microwave power was considered to be totally absorbed by a water load at the end of the waveguide (Figure 2B). In reality, a small amount of this wave is reflected by the water load, allowing for the consideration that the input microwave power ($P_{in}$) in the empty rectangular cavity can be calculated according to Equation (1), where $\dot{m_{w}}$ is the mass flow rate of water (7.84 ± 0.09 g/s), $C_{p,w}$ is the average specific heat capacity of the water [42], and ΔT is the difference between the inlet and outlet temperatures of the water stream.

(1) $$P_{in}=\dot{m_{w}}\;C_{p,w}\;\Delta T$$

### 2.2. Numerical Model Development 

#### 2.2.1. Thermo-Physical and Dielectric Properties 

Accurate thermo-physical properties of the food under the processing conditions are important input parameters for the simulation [43]. For microwave heating, dielectric properties of the food are fundamental as well. Herein, experimental measurements of density (ρ), specific heat ($C_{p}$), thermal conductivity (k), relative dielectric constant ($\varepsilon^{\prime }$), and relative dielectric loss factor ($\varepsilon^{\prime \prime }$), as a function of temperature, were performed for cooked peeled shrimp paste. 

A batch of frozen and peeled shrimp (5 kg, initial temperature ≤ 2 °C) was immersed in a hot water bath at 95 °C and kept for 1 min after the core temperature reached 75 °C. This temperature was chosen to meet the Guide on Good Hygiene Practices [44] for cooking. Then, the samples were immersed in an iced water bath until the core temperature reached 2 °C (less than 30 min). The core temperature was monitored with a type-K thermocouple placed at the second abdominal segment of two shrimp and recorded every second using TH-060 data logger (Instrutherm, São Paulo, Brazil). After chilling, 200 cooked peeled shrimp were packaged in plastic bags and stored at 4 °C for experimental validation using a cylindrical applicator. The remaining portion of the cooked peeled shrimp was turned into a paste using a 2P domestic chopper (Britânia, Curitiba, Brazil) before the determination of thermo-physical and dielectric properties. This was necessary to provide a homogeneous material for properties determination.

A cylindrical metal pycnometer (25 mL) was used to determine the density (ρ) according to Rahman [43]. The filled pycnometer was placed in a RE 620S thermostatic bath (Lauda Scientific GmbH, Lauda-Königshofen, Germany) at the desired temperature for at least 30 min. After temperature equilibrium, the excess of the sample was removed. The pycnometer was then weighed using JK-200 analytical balance (Chyo, Kyoto, Japan) at room temperature. The density was determined in triplicate at 3, 25, 50, 75, and 90 °C. 

Thermal properties (thermal conductivity k and specific heat $C_{p}$) were measured using KD2 Pro Thermal Properties Analyzer with SH-1 sensor (Decagon Devices, Pullman, WA, USA). Measurements of the thermal properties were carried out after keeping cylindrical flasks filled with 25 g of samples for 30 min in a RE 620S thermostatic bath (Lauda Scientific GmbH, Lauda-Königshofen, Germany) at selected temperature (3, 25, 50, 75, and 90 °C). The experimental determination of the thermal properties was performed in triplicate for each temperature. 

Relative dielectric constant ($\varepsilon^{\prime }$) and relative dielectric loss factor ($\varepsilon^{\prime \prime }$) were determined by reflection measurements from E5061B Network Analyzer (Agilent Technologies, Bayan Lepas, Malaysia) with an open-ended coaxial-line high-temperature probe (Agilent Technologies, Bayan Lepas, Malaysia) at the frequency range of 200 to 3000 MHz with 100 linearly spaced points. To provide a reliable determination of the dielectric properties from the measured reflection coefficients, we warmed the system up for 90 min before the calibration procedure with air (open circuit), short circuit, and deionised water. For measurements at different temperatures (3, 25, 50, 75, and 95 °C), a stainless-steel jacketed vessel (38.45 mm of outside diameter and 21.65 mm of inside diameter) with a MT-455 thermocouple (Minipa, Joinville, Brazil) on a bottom well was used. The jacketed vessel was filled with shrimp paste and connected to a TC500 thermostatic oil bath (Brookfield, Middleborough, MA, USA). The dimensions of this custom-made vessel were selected to avoid interference with the dielectric measurement of food products. Dielectric properties were measured in triplicate at a given temperature and the experimental procedure was carried out twice. 

#### 2.2.2. 3D Geometric Representation of the Cooked Peeled Shrimp

To better understand the microwave heating of the shrimp, we limited the study to a single un-packed, cooked, and peeled shrimp. Considering that the 3D construction of the external contours is enough for good accuracy of the numerical model, the 3D representation of a shrimp was obtained in a stereolithography (STL) file from a model repository and imported into COMSOL Multiphysics software (v. 5.5, COMSOL Inc., Stockholm, Sweden) [45]. 

**Figure 3:**
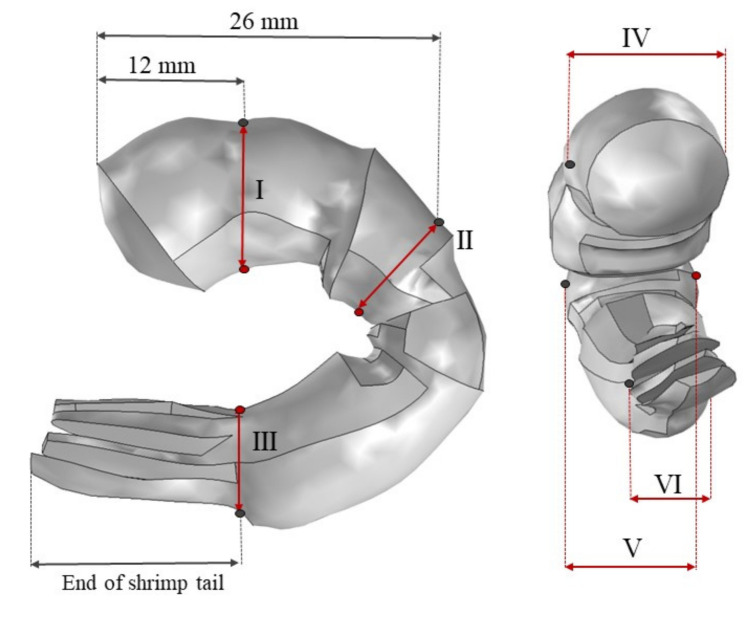
Representation of six different length measurement locations of the shrimp sample and 3D model representation.

To approximate the 3D model to real shrimp dimensions, we measured the external dimensions at six different locations (points I, II, III, IV, V, and VI) using a calliper with an accuracy of ± 0.03 mm, according to Figure 3. One tray of ready-to-eat shrimp (130 g of cooked and peeled shrimp) was purchased at a local market in Nantes (France). Average external dimensions were obtained from the measurement of 10 cooked peeled shrimp before and after microwave heating (rectangular waveguide cavity at $P_{in\;}$= 40 W and Δt = 100 s). The original 3D model of the shrimp was scaled using COMSOL Multiphysics software (v. 5.5, COMSOL, Stockholm, Sweden) to better fit the real dimensions of the shrimp. 

#### 2.2.3. General Assumptions and Governing Equations 

The numerical study of microwave heating of a solid food consists of coupling and solving Maxwell’s equations of electromagnetism and the general heat transfer equation to predict the electromagnetic field propagation and the temperature distribution within the product. For this purpose, the assumed conditions were:

(A1) Food is non-magnetic ($\mu_{r}$ = 1), homogeneous, and isotropic (thermo-physical and dielectric properties of the shrimp paste).
(A2) Uniform distribution of the initial temperature in the food (T = 20 °C at t = 0 s).
(A3) Dependence of thermo-physical and dielectric properties with temperature (ρ(T), $C_{p}$(T), k(T), $\varepsilon^{\prime }$(T), and $\varepsilon^{\prime \prime }$(T) for 3 °C ≤T≤ 90 °C) according to the adjusted correlations.
(A4) No shrinkage or swelling of the product during microwave treatment as well as no mass transfer such as water loss.
(A5a) Negligible interactions between the PTFE and Pyrex supports and electric field.
(A5b) Thermal resistance of the PTFE and Pyrex supports was disregarded.
(A6) Perfect electrical conductors ($\vec{E}\times \vec{n}\;$= 0) in the rectangular and cylindrical cavity walls.
(A7) Constant air temperature in the cavity ($T_{ext\;}$= 20 °C, ∀ t ≥ 0 s).
(A8) Shrimp surrounded by air (zero dielectric loss) and with heat losses by natural convection ($-\vec{n}\left(-k\nabla T\right)=-h\left(T-T_{ext}\right)$) with heat transfer coefficient (h) evaluated for a vertical thin cylinder (height = 2.10 cm and diameter = 0.81 cm).
(A9a) The wave reflection at the end of the rectangular waveguide (water load) is negligible.
(A9b) Circular opening to air in the top of the cylindrical applicator.

The coupling of the first (Ampere’s law) and the second (Faraday’s law) of Maxwell’s equations, assuming both time-harmonic fields in a homogeneous source-free medium, is expressed in Equation (2):

(2) $$\nabla^{2}\vec{E}+\varepsilon {\left(\frac{2\pi f}{c}\right)}^{2}\vec{E}=\vec{0}$$

where $\vec{E}$ is the electric field strength (V/m), f is the frequency (Hz), c is the speed of light (3∙10^8^ m/s), and $\varepsilon =\varepsilon^{\prime }-j\varepsilon^{\prime \prime }$ is the relative complex permittivity [27].

The heat transfer inside the solid-food product was modelled by the generalised heat equation with a source term, as in Equation (3) [27]:

(3) $$\rho C_{p}\frac{\partial T}{\partial t}=div\left(k\nabla T\right)+Q_{gen}$$

(4) $$Q_{gen}=\frac{1}{2}\;\omega \varepsilon_{0}\varepsilon^{\prime \prime }{\left|\vec{E}\right|}^{2}$$

(5) $$P_{abs}=∭Q_{gen}dV$$

where the last term of Equation (4) represents the volumetric heat generation ($Q_{gen}$) by microwave radiation from the local electric field strength (W/m^3^), and $P_{abs}$ is the microwave power dissipated inside a volume (V).

The absorbed microwave power of each applicator was calculated from the simulation by volume integration of the sample (Equation (5)) and compared at the same emitted microwave power (40 W).

#### 2.2.4. 3D Meshing Procedure and Numerical Resolution

The simulation of the electromagnetic wave propagation (Equation (2)) and heat transfer (Equation (3)) was performed by COMSOL Multiphysics software (v. 5.5, COMSOL, Stockholm, Sweden) using the Heat Transfer and Radiofrequency modules. Numerical simulations were performed in a Dell Precision T7810 workstation (Dell Inc., Round Rock, TX, USA) with Intel Xeon processor at 2.56 GHz and 256 GB of RAM (Intel Corporation, Santa Clara, CA, USA). Total computation times required for the rectangular and cylindrical applicators systems were around 2 min and 7 min, respectively. 

The tetrahedral element was used as the basic element for the volume mesh on the basis of previous works [40,45]. For the rectangular applicator, the mesh consisted of 6976 tetrahedral elements (“finer” size) with max. element size: 6.91 mm and min.: 0.86 mm for all domains (shrimp, air, and brass). The mesh for the cylindrical applicator system was set as “coarser” (max. element size: 12.7 mm and min.: 3.80 mm) for the shrimp domain, “extremely coarse” (max. element size: 41.8 mm and min.: 8.87 mm) for the other domains (air and PTFE). There was observed the mesh independence of numerical results for the selected mesh size from 97671 (“coarser”) to 1430657 (“fine”) tetrahedral elements in the cylindrical applicator. 

### 2.3. Experimental Validation 

**Figure 4:**
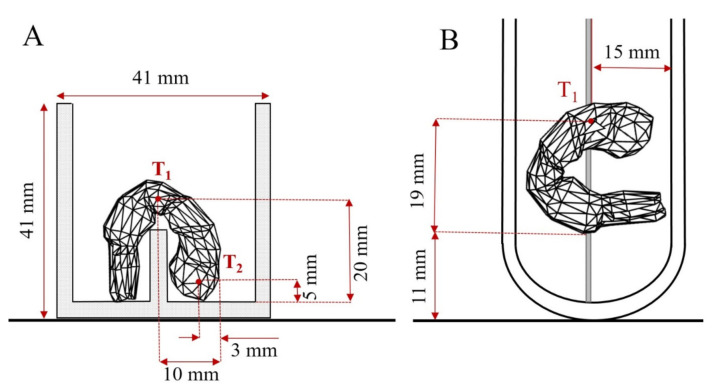
Sample holder dimensions with fibre optic sensor positions (T_1_ and T_2_) used to perform microwave heating in the rectangular cavity system (**A**) and commercial microwave device (**B**).

To check the validity of the numerical models, we performed the direct temperature measurements at selected locations during microwave heating (Figure 4) using fibre optic temperature sensors, and data were compared with the simulation results. For both applicators, the sample holder was neglected in the modelling approach, as its influence on the reflection coefficient was small (less than 2 %).

To validate the mathematical model of the rectangular applicator, we purchased two trays of cooked and peeled shrimp (260 g) at a local market in Nantes (France). A single shrimp was selected, weighted, and heated by placing it in a holder structure at the centre of the rectangular applicator. To ensure the same sample position, we custom-made a PTFE holder structure comprised of a slab (84 × 41 × 11 mm) and case (41 × 41 × 11 mm). The PTFE support had holes for introducing the fibre optic sensors at different locations, maintaining the exact location of the probes in the shrimp. The tips of the temperature sensors were inserted at the centre of the shrimp at two different positions, as shown in Figure 4A. Temperatures of the samples were recorded every second using a fibre optic thermometer (Neoptix, Quebec City, Canada) connected to a ReFlix-4 data logger (Neoptix, Quebec City, Canada), starting at a room temperature of around 20 °C. The experiments were performed in quadruplicate at three combinations of power level ($P_{in}$) and heating time (t), such as 11 W/110 s, 32 W/35 s, and 43 W/20 s. 

For the experimental validation using cylindrical applicator, the samples preparation was carried out as the description in Section 2.2 following the Guide of Good Hygiene Practices [44]. A Pyrex reaction vessel (110 mm of height and 30 mm of inner diameter) was placed in the centre of the cylindrical applicator cavity. The temperature of the food sample was measured using a fibre optic probe placed at the centre of the second abdominal section of the shrimp, as shown in Figure 4B. Ideally, for a suitable evaluation of temperature distribution during microwave heating, several temperature sensors should be placed inside the food; however, this was not possible due to the limitation of the reaction vessel dimensions. Temperatures were recorded every 0.5 s using a fibre optic thermometer Luxtron 812 (LumaSense Technologies, Santa Clara, CA, USA). The microwave heating experiments were carried out at three combinations of nominal power levels (P) and heating time (t), 50 W/18 s, 75 W/10 s, and 150 W/5 s, in quadruplicate.

### 2.4. Statistical Analysis

To evaluate influence of the microwave heating on dimensions and mass of shrimp before and after treatment, the analysis of variance (one-way ANOVA) followed by Tukey’s test (p≤ 0.01) were carried out with Minitab software (v. 19, Minitab Inc., State College, PA, USA). 

Dielectric properties ($\varepsilon^{\prime }$ and $\varepsilon^{\prime \prime }$) at 2450 MHz were calculated by linear interpolation of adjacent points and correlated with temperature (3 °C ≤T≤ 95 °C) using the multiple regression tool of Excel software (v. 2016, Microsoft, Redmond, WA, USA). Similarly, empirical correlation of thermo-physical properties (ρ, $C_{p}$, and k) with temperature were adjusted. Electrical conductivity (σ) was calculated on the basis of dielectric loss factor ($\varepsilon^{\prime \prime }$) at 2450 MHz, as described in Equation (6), where ω = 2πf is the angular frequency of the electromagnetic waves (rad/s) and $\varepsilon_{0}$ is the permittivity of free space (8.854 × 10^−12^ F/m) [46].

(6) $$\sigma =\omega \varepsilon_{0}\varepsilon^{\prime \prime }$$

To assess the numerical simulation of the model, we compared the predicted time-temperature profiles to experimental profiles using the root mean square error equation (RMSE), as shown in Equation (7):

(7) $$RMSE=\sqrt{\frac{1}{N}\overset{N}{\underset{i=1}{\sum }}{\left(T_{pred}-T_{exp}\right)}^{2}}\;$$

where N is the number of data points (N = 12), and $T_{pred}$ and $T_{exp}$ are the predicted and the experimental temperatures at a specific probe location (°C), respectively. For each probe location, the mean of the four experiments was used.

## 3. Results and Discussion

### 3.1. Properties of the Food Material 

The mean values of dimensions and mass of the cooked peeled shrimp samples and 3D scaled model are presented in Table 1 and Figure 3. Although the values of mass before and after microwave heating were not statistically different, three dimensions (I, II, and IV) had a small reduction after microwave treatment (p≤ 0.01). 

**Figure 5:**
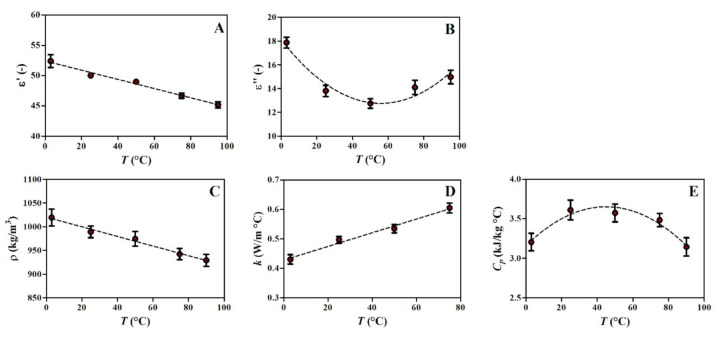
Relative electrical permittivity at 2450 MHz (**A**), relative dielectric loss factor at 2450 MHz (**B**), density (**C**), thermal conductivity (**D**), and specific heat capacity (**E**) of shrimp paste.

The effect of temperature (3 °C ≤T≤ 95 °C) on relative electrical permittivity ($\varepsilon^{\prime }$) and relative dielectric loss factor ($\varepsilon^{\prime \prime }$) of shrimp paste at 2450 MHz is shown in Figure 5A,B. As temperature increased (i.e., higher thermal agitation), the relative electrical permittivity decreased since the free water molecules of the food material had more difficulty to align to the electric field [47].

Empirical correlations of thermo-physical and dielectric properties of shrimp paste at 2450 MHz in the temperature range of 3 to 95 °C are given in Table 2 and shown in Figure 5. It should be noted that the classical prediction equations based on major food constituents proposed by Choi and Okos [48] have a positive linear relationship of thermal-physical properties with temperature, whereas the empirical correlations in Figure 5B,E were second-order polynomial models. Advancements in instrumentation, such as the dual needle line heat source, controlled temperature vessel, and accurate temperature sensor, have provided a more accurate non-linear dependency of thermo-physical properties of food products with temperature over the last two decades [49]. Similar values of thermo-physical properties of shrimp can be found in the literature [24,50]; although, an accurate prediction as a function of temperature is of great importance for a reliable numerical simulation. 

### 3.2. Electromagnetic Field Distribution in the Cavities

Spatial distribution of the electromagnetic field depends on the dielectric properties and geometry of the food, as well as the geometric structure of the cavity [49]. Empty single-mode microwave applicators with simple geometrical shape (e.g., rectangular or cylindrical) have a well-defined electric field pattern [24,51]. 

**Figure 6:**
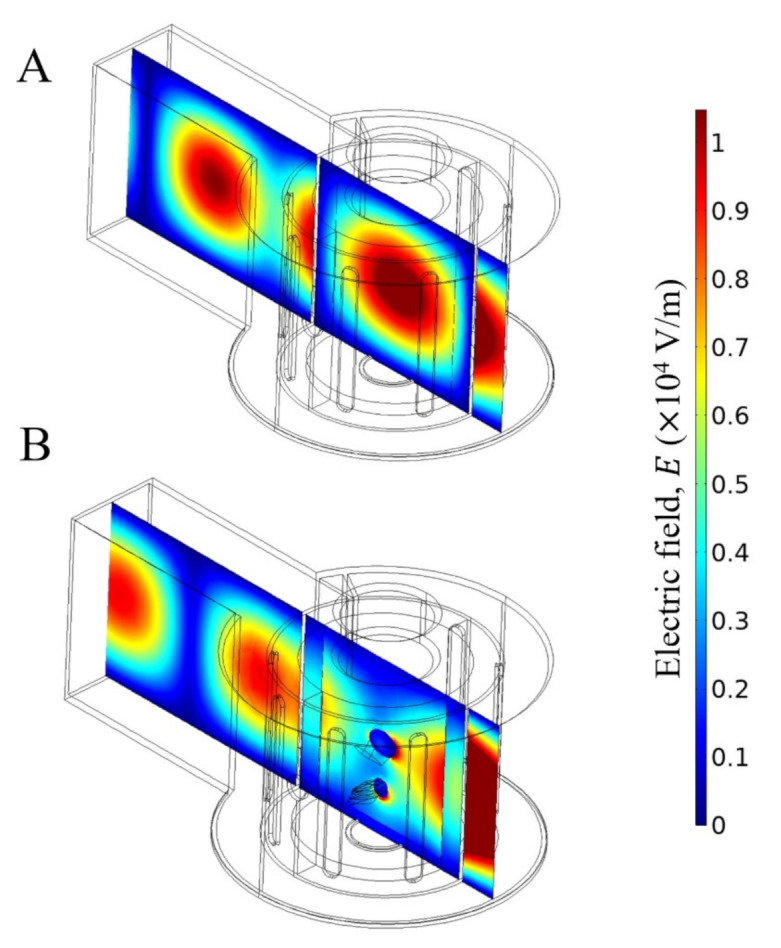
Electric field distribution within an empty cylindrical cavity (**A**) and shrimp loaded cavity (**B**). Incident microwave power = 40 W and frequency = 2450 MHz.

**Figure 7:**
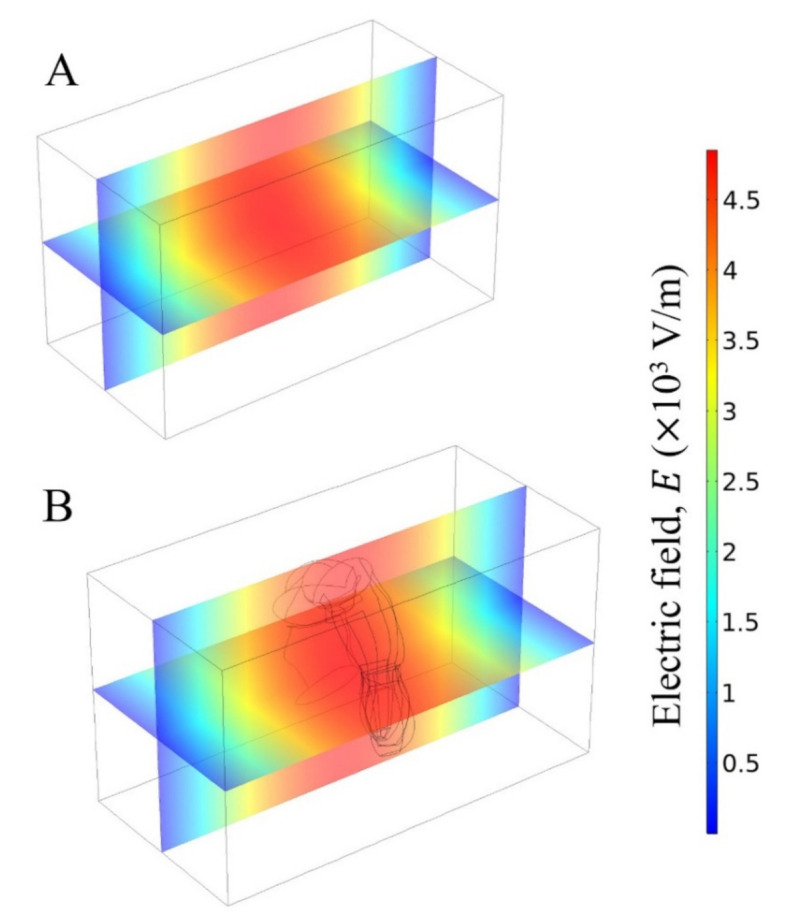
Electric field distribution inside the empty rectangular cavity (**A**) and when the shrimp was placed in the centre (**B**). Incident microwave power = 40 W and frequency = 2450 MHz.

As a starting point, the electromagnetic modelling was verified according to the similarity principle between the applicator geometry and spatial field distribution within the empty microwave applicators at 2450 MHz (Figure 6A and Figure 7A). Measuring and validating transient electric field distribution in 3D food product is still a particular challenge [52]. One convenient solution is to compare the electric field distribution within microwave applicator with fundamental waveguide theory (Figure 1). Both applicators presented a good agreement between spatial electromagnetic field distribution and geometrical shape, as reported in previous studies [19,25,41]. For this reason, the shrimp was placed in the centre of the rectangular and cylindrical cavities for the experiments, where the maximum intensity of the electric field was observed.

When the microwave applicator was loaded (Figure 6B and Figure 7B), there were some perturbations of the original electric field intensity for cylindrical and rectangular applicators. For the same emitted microwave power (P =$\;P_{in}$ = 40 W), the power absorbed by the shrimp was 15 W for the cylindrical applicator, while it was 18 W for the rectangular applicator, resulting in different heating rates. In the studied microwave applicators, there are no adjustment devices to obtain the minimum reflected power from the source. As a consequence, the absorbed and reflected powers were different for each microwave applicator. 

### 3.3. Temperature Distribution from Numerical Simulation and Experiments 

**Figure 8:**
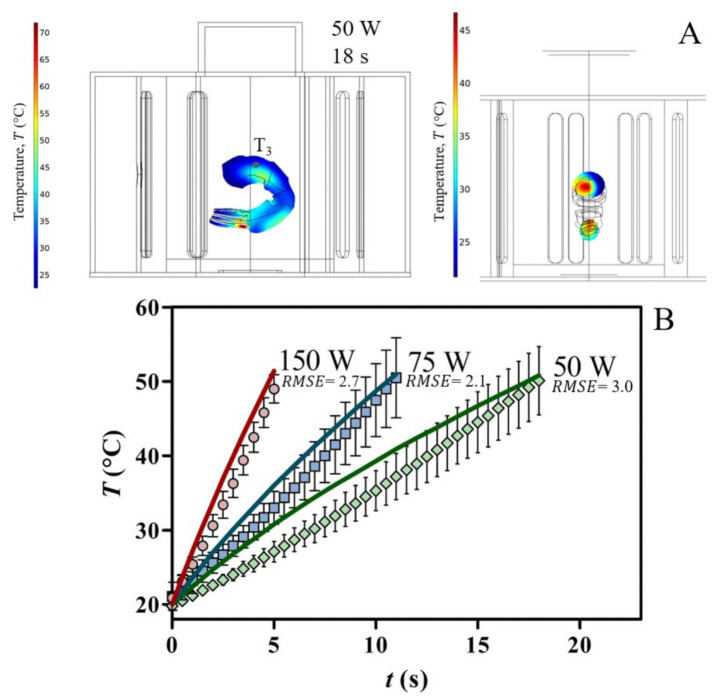
Computed heating pattern in the shrimp sample loaded into cylindrical applicator after 18 s at 50 W (**A**). Evolution of experimental and simulated temperatures of shrimp sample when varying the microwave power and heating time (**B**).

**Figure 9:**
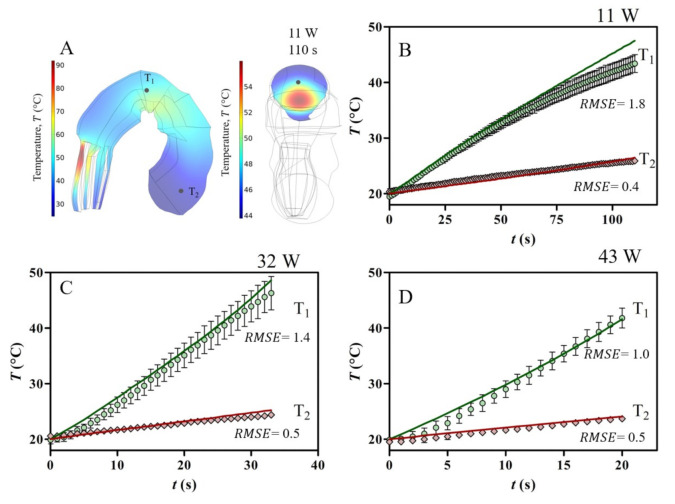
Computed temperature distribution in the shrimp sample loaded into rectangular applicator after 110 s at 11 W (**A**). Experimental and computed temperature histories of shrimp sample at 11 W/110 s (**B**), 32 W/35 s (**C**), and 43 W/20 s (**D**).

The plots in Figure 8 and Figure 9 show the predicted temperature evolution in the shrimp compared with temperatures experimentally measured in different locations, as defined in Figure 4, at different power level settings. In the experiments, we noticed a high sensitivity to probe position, resulting in elevated standard deviations (≤ 7 %). The inhomogeneous temperature distribution pattern at the end of heating is evidenced in Figure 8A and Figure 9A, and it was observed regardless of the power setting.

As an example, applying an input power of 50 W in the cylindrical applicator, the highest temperature difference was 55 °C after 18 s of heating due to the microwave focusing effect at the shrimp tail (not experimentally validated). Disregarding the shrimp tail, the highest temperature difference in the abdominal section was around 35 °C after 18 s (Figure 8A). For the rectangular applicator (Figure 9A), the numerical simulation also predicts the same trend of temperature distribution after 110 s at 11 W. 

Significant temperature gradients in solid foods inside a rectangular single-mode microwave device with constant input power were previously reported in the literature [3,19,53]. In the cylindrical applicator, the cross-sectional temperature gradient of shrimp showed a high-temperature region displaced from the geometric centre of the sample (Figure 8A), which is consistent with the field distribution in Figure 6B. In this case study, selecting the rectangular applicator can decrease the temperature difference in the direction of the thickness (ΔT = 10 °C) compared to the cylindrical applicator (ΔT = 20 °C). These results highlight the problem of determining hot/cold zones for suitable microwave thermal processing of solid foods. 

Figure 8B and Figure 9B show a good agreement between predicted and measured temperatures. Among other factors, the correlations between temperature and properties of shrimp paste (Table 2) are valid for these tested conditions. As reported in previously studies [25,40], the uncertainty of temperature probe position and small variations of the sample dimensions could cause significant temperature discrepancies between predicted and experimental data points, mainly in small samples. 

Due to the fact that the electromagnetic field is sensitive to modifications in geometry (i.e., sample size) and thermal and dielectric properties (i.e., $C_{p}$, k, $\varepsilon^{\prime }$, and $\varepsilon^{\prime \prime }$) combined with the variation of shrimp dimensions/properties by the farming conditions and/or breed, it is still a challenge to propose a general microwave system for thermal treatment of solid food that integrate these biological variances [54]. The findings of this study highlight two important points related to microwave simulation. First, the real shape of food has a substantial impact on the temperature distribution during microwave processing. Second, the development of mathematical model coupling electromagnetic equations and heat transfer is of great importance for an appropriate evaluation of food heating. Such approach is recommended for microwave heating of solid foods, due to the difficult prediction of temperature distribution as a consequence of volumetric heating and non-uniform electromagnetic field within a loaded single-mode cavity.

## 4. Conclusions

In this study, the influence of two lab-scale microwave applicators on the temperature distribution of cooked peeled shrimp was compared using a 3D mechanistic model. The numerical simulation provided the electromagnetic field pattern and the temperature distributions in the shrimp as a function of time. Spatial field distribution showed a high sensitivity to input power values, which makes the prediction of hot/cold zones in solid food a not trivial task. Despite this high sensitivity degree during the experimental trials, temperature evolutions at different locations were compared and presented good agreement with predicted temperatures (RSME≤ 3.0) for both microwave applicators. The lower temperature gradients were observed in the cross-section of the shrimp heated in a rectangular single-mode device compared to cylindrical cavity. This result indicates that for similar sample sizes and microwave input powers, the geometrical configuration of microwave cavity is of great importance. However, it is not enough for overcoming the non-uniform temperature distribution in solid foods. Considering that microwave cavity design can change the electromagnetic field pattern and temperature distribution within the solid food, this model needs to be extended for the thermal treatment of a larger batch of samples (i.e., packaged shrimp moving in the microwave cavity). Moreover, this numerical approach can provide future directions for the choice of a suitable configuration of microwave system according to a specific purpose, such as shelf-life extension of cooked peeled shrimp.

## Figures and Tables

**Table 1:** Values of mass (g) and external dimensions (mm) of the samples and 3D model at six different positions (Figure 3).

	Dimensions (mm)						Mass (g)
	I	II	III	IV	V	VI	
Before heating	11.6 ^a^ ± 0.5	10.2 ^a^ ± 0.8	6.89 ^a^ ± 0.57	9.07 ^a^ ± 0.77	6.86 ^a^ ± 0.10	4.60 ^a^ ± 0.81	4.07 ^a^ ± 0.35
After heating and cooling	10.4 ^b^ ± 0.9	9.12 ^b^ ± 0.32	6.67 ^a^ ± 0.51	6.63 ^b^ ± 0.78	5.77 ^a^ ± 0.59	4.13 ^a^ ± 0.16	3.53 ^a^ ± 0.45
3D model	8.57	5.11	5.90	8.83	8.01	5.45	3.79

Results are mean of six repetitions ± standard deviation. In columns, different letters denote significant differences (p≤ 0.01) according to Tukey’s test.

**Table 2:** Adjusted polynomial correlations for the temperature dependence (3 °C ≤T≤  95 °C) of thermo-physical and dielectric properties of shrimp paste.

Property	Polynomial Correlations	$R^{2}$
Thermal conductivity (W/m °C)	$k=2.32\times 10^{-3}T-0.43$	0.985
Specific heat capacity (kJ/kg °C)	$C_{p}=-2.42\times 10^{-4}T^{2}+2.18\times 10^{-2}T+3.16$	0.938
Density (kg/m^3^)	$\rho =-1.02\;T+1.02\times 10^{3}$	0.987
Relative dielectric constant at 2450 MHz (-)	$\varepsilon^{\prime }=-7.59\times 10^{-2}T+52.4$	0.986
Relative dielectric loss factor at 2450 MHz (-)	$\varepsilon^{\prime \prime }=1.71\times 10^{-3}T^{2}-1.91\times 10^{-1}\;T+18.1$	0.926

Parameters of the polynomial regressions are means of three repetitions for the thermal properties and six repetitions for the dielectric properties.

## Data Availability

The datasets generated for this study are available on request to the corresponding author.

## Nomenclature

$C_{p}$	specific heat capacity	(J/kg·°C)
c	speed of light	(m/s)
$\vec{E}$	electric field vector	(V/m)
$E_{0}$	incident electric field amplitude	(V/m)
f	frequency	(Hz)
$\vec{H}$	magnetic field vector	(A/m)
h	heat transfer coefficient	(W/m^2^·°C)
k	thermal conductivity	(W/m·°C)
m	mass	(g)
$\dot{m}$	mass flow rate	(g/s)
N	number of data points	(-)
$\vec{n}$	unit vector normal to the boundary surface	(-)
P	microwave power	(W)
Q	volumetric heat	(W/m^3^)
RSME	root mean square error	(°C^2^)
T	temperature	(°C)
t	time	(s)
V	volume	(m^3^)
**Greek Symbols**		
ε	relative complex permittivity	(-)
$\varepsilon^{\prime }$	relative dielectric constant	(-)
$\varepsilon^{\prime \prime }$	relative dielectric loss factor	(-)
$\varepsilon_{0}$	permittivity of free space	(F/m)
$\mu_{r}$	relative magnetic permeability	(H/m)
ρ	material density	(kg/m^3^)
σ	electrical conductivity	(S/m)
ω	angular frequency	(rad/s)
**Subscripts**		
abs	absorbed	
exp	experimental	
ext	external ambient air	
gen	generated	
in	input	
pred	predicted	
w	water	
